# A Commentary on Telerehabilitation Services in Pakistan: Current Trends and Future Possibilities

**DOI:** 10.5195/ijt.2017.6224

**Published:** 2017-06-29

**Authors:** ZESHAN ZAHID, SULEMAN ATIQUE, MUHAMMAD HAMMAD SAGHIR, IFTIKHAR ALI, AMNA SHAHID, REHAN ALI MALIK

**Affiliations:** 1 HEALTH INFORMATICS UNIT, COMSATS INSTITUTE OF INFORMATION TECHNOLOGY, ISLAMABAD, PAKISTAN; 2 DEPARTMENT OF MEDICAL INFORMATICS, TAIPEI MEDICAL UNIVERSITY, TAIPEI, TAIWAN; 3 DEPARTMENT OF COMPUTER SCIENCES, UNIVERSITY OF ENGINEERING AND TECHNOLOGY, LAHORE, PAKISTAN; 4 DEPARTMENT OF PHYSIOTHERAPY, RIPHAH UNIVERSITY, ISLAMABAD, PAKISTAN; 5 DEPARTMENT OF ECONOMICS, PUNJAB UNIVERSITY, LAHORE, PAKISTAN; 6 DEPARTMENT OF ORTHOTICS & PROSTHETICS, RAWALPINDI MEDICAL COLLEGE, RAWALPINDI, PAKISTAN

**Keywords:** eHealth, mHealth, Pakistan, Telemedicine, Telehealth, Telerehabilitation

## Abstract

A 2014 World Health Organization (WHO) study reported that almost 27 million people with disability live in Pakistan with fewer than one allied rehabilitation professional per 10,000 people. The current study sought to determine the attitudes toward telerehabilitation via a survey administered to 329 Pakistani rehabilitation professionals. Study results indicate that rehabilitation professionals in Pakistan are knowledgeable about telerehabilitation and Information and Communication Technology (ICT), and are receptive to employing telerehabilitation programs and applications. Therefore, we can infer that the future of telerehabilitation can be bright in Pakistan but requires the attention of policy makers and non-government organizations to launch an appropriate program nationwide. The authors suggest that a range of telerehabilitation services (e.g., consultation, assessment, and therapy) could alleviate the shortage of rehabilitation personnel in Pakistan.

## BENEFITS OF TELEREHABILITATION

Telerehabilitation can provide synchronous and comparatively low cost but quality rehabilitation services regardless of time, space, and location (Eron, 2010). Telerehabilitation can be a viable alternative for persons with disabilities who are unable to physically visit facility based rehabilitation centers (Tyagi, Aikat, & Singh, 2016).

Indeed, the future of telerehabilitation seems to be bright due to its flexibility, remote availability, and cost effectiveness. The use of telemedicine provides patients and their practitioners with opportunities to consult with the most experienced and expert practitioners world-wide (dos Santos et al., 2014). Telerehabilitation can be used to improve Community Based Rehabilitation (CBR) systems, particularly in developing countries where rehabilitation services are very expensive and not available at the community level (Kairy, Lehoux, Vincent, & Visintin, 2009).

Telerehabilitation is in practice for the care of older persons. There is evidence that telerehabilitation can foster the resumption of activities of daily living of older persons and therefore can improve their life style and social integration (Gregory, Alexander, & Satinsky, 2011). Recent research also shows high satisfaction from the users in a balance training program with significant effects on mobility and balance respectively.

Telerehabilitation may be a practical alternative to conventional (i.e., in-person) rehabilitation therapy for patients with total knee arthroplasty (Shukla, Nair, & Thakker, 2016). Other research found that telerehabilitation can foster continuity of therapy in the home; patients demonstrate improved compliance and physical ability (Reinkensmeyer, Pang, Nessler, & Painter, 2002).

Evidence shows that telerehabilitation robotic devices are able to extend effective and evidence based specialized rehabilitation services for upper and lower limb rehabilitation to rural veterans with poor access to health and rehabilitation care (Cherry et al., 2017).

The literature shows that telemedicine is a cost effective and efficient method to deliver community based health care services. A study in Ghana suggested that there was 80% reduction in the overall cost of primary health care when delivered through telemedicine. The study respondents also suggested that telerehabilitation methodology can be used to provide rehabilitation services to achieve WHO goals (e.g., improved access to health care services and professionals) (Otsen & Agyei-Baffour, 2017). Telemedicine has proven to be an effective tool to monitor, surveil, standardize, and implement health care services, as well as to conduct research and development (Schutte et al., 2012).

## REHABILITATION NEEDS IN PAKISTAN

There is a severe shortage of rehabilitation professionals (e.g., physiotherapists, occupational therapists, speech-language pathologists, psychologists, orthotists and prosthetists, rehabilitation counselors, and rehabilitation nurses) in Pakistan with less than one rehabilitation professional for every 10,000 people (Gupta, Castillo-Laborde, & Landry, 2011). The same is true for India, Bangladesh, and Sri Lanka.

Seventy per cent of the Pakistani population live in rural areas. In contrast, only 22% of Pakistani physicians reside in rural locations. Telehealth programs could be helpful to rural areas deprived of basic health care and rehabilitation services. Thus, there is a strong stimulus to initiate telerehabilitation programs in Pakistan.

The World Health Organization (WHO, 2014) developed a global disability action plan for 2014–2021 to ensure the availability of health care and rehabilitation services for persons with disabilities, especially those in developing countries. The WHO estimated that though almost 27 million people in Pakistan live with a disability, only 23 physical medicine specialists practice in Pakistan.

Though Pakistan enjoys a greater availability of rehabilitation services than many other countries in the region, only three rehabilitation centers and fifteen departments of rehabilitation medicine operate across the country (Rathore, New, & Iftikhar, 2011). Additionally, only four regional facilities and two functional physical medicine and rehabilitation institutes provide rehabilitation services in Pakistan. One of these institutes is administered by the Pakistani army and the other operates in the private sector. Most of these facilities do not offer a multidisciplinary approach because of the scarcity of rehabilitation professionals and the operational costs (Khan et al., 2017). Data concerning the exact number of rehabilitation centers and professionals in Pakistan is unknown since there is no central data source.

## TELEHEALTH IN PAKISTAN

Telehealth is currently used in Pakistan, yet exercises a negligible impact on the healthcare system. Telehealth was initiated as a pilot project in Pakistan in 2001 by the Commission on Science and Technology for Sustainable Development in the South (COMSATS) in Gujar Khan. In 2004 COMSATS opened a second telehealth center in Skardu in collaboration with the Canadian International development research center (Hussain, 2007). In 2011 COMSATS opened a third telehealth center in Zhob, Baluchistan.

In the last few years the rehabilitation system in Pakistan has improved to some degree, and is comparatively better than other SAARC countries. However, the use of telerehabilitation still needs more consideration, especially in rural areas. Telerehabilitation is also poorly integrated with the country’s acute care system (Khan et al., 2017)

The All India institute of Speech and Hearing (AIISH) operates a videoconferencing center for rehabilitation and distance learning and offers a diploma in hearing, language, and speech. The private sector in India is also contributing to these areas, and there is a service provider called “Vaibhav Pvt Ltd” from Chennai providing telerehabilitation services. In Bangladesh there is a telerehabilitation program for hand skill development for the disabled community (Uddin, Khan, & Mahmud, 2012). However, in Pakistan there is no such telerehabilitation program in practice thus far.

Despite the successes of these efforts, it was unclear whether Pakistan-based professionals are sufficiently knowledgeable about Information and Communication Technology (ICT) and telerehabilitation to feel confident in using this service delivery model. The purpose of this study was to explore Pakistan-based professionals’ knowledge of ICT and their confidence in using telerehabilitation.

## SURVEY METHODOLOGY

The authors administered a survey to 329 rehabilitation professionals across Pakistan. Professionals who worked in various government, private, and charitable facility based rehabilitation centers were recruited using convenience sampling.

Initially, the questionnaire contained both open-ended and closed-ended questions, with the Likert scale employed for the closed-ended questions. In a pilot study, the questionnaires were administered to ten different rehabilitation professionals with at least 15 years of experience in their respective field. While all participants registered responses for the closed end questions, most left the open-ended questions unanswered. Therefore, the open-ended questions were removed from the questionnaire.

A revised questionnaire was distributed among 329 rehabilitation professionals. While participants were initially given seven days to return the questionnaire, the response window was extended for an additional week due to an initially low response rate. In total, 310 participants returned the questionnaire, an excellent response rate. Of these, twenty-three questionnaires were rejected due to rejection criteria that included a failure to answer all questions, selection of more than one option, and/or missing demographic information (i.e., gender and professional information).

Participants completed a consent form attesting that they would provide valid information according to the best of their knowledge. The researcher provided participants with written assurance that their personal information would not be shared with others.

## SURVEY RESULTS

Table 1 shows characteristics of the study participants.

The survey data were first coded and then analyzed using SPSS version 16 (Corp, 2007); descriptive statistics were calculated. Results are presented in Table 2.

Fifty-one percent had good ICT knowledge, in that they owned personal computers and used the internet on a regular basis. Almost 91% of the participants were familiar with the communication tool Skype and used Skype to make video calls for patient consultations and online meetings. Participants were asked about their knowledge about telemedicine technology: 41% have not used the technology but were aware of it; 31% indicated they were aware of the technology and were using it on an intermittent basis; 14% were using the technology on a regular basis; and 24% were not familiar with the technology.

Regarding telerehabilitation, 35% of participants reported they didn’t use the technology but were aware of it; 34% were aware of the technology and used it on an intermittent basis; 9% were fully aware of the technology and used it on a regular basis; and 22% were not aware of the technology. To improve access to care, 23% participants recommended telerehabilitation as a potential solution. Thirty-eight percent of participants reported that rehabilitation services in Pakistan could be improved via the use of Community Based Rehabilitation (CBR). Thirty-nine percent of participants preferred the use of both telerehabilitation and CBR programs to improve the service delivery system.

More than 68% of the participants affirmed that with the help of telerehabilitation, a variety of services including consultation, prescription, delivery of required therapy, monitoring, and nursing care can be provided. More than 27% said that telerehabilitation should only be employed for consultation, prescription, delivery of required therapy or monitoring.

When participants were asked about the social acceptance of telerehabilitation in Pakistan, 21% said that telerehabilitation will be an “extremely acceptable” service, and 35% rated is as “very acceptable.” When asked to identify potential limiting factors for telerehabilitation use in Pakistan, more than half of the participants selected a lack of ICT knowledge, high cost, rapidly changing ICT technology, attitude of policy makers toward telerehabilitation programs, lack of skilled personnel, and patient compliance as challenges.

A majority of the participants suggested that an individual patient may be exposed to some risks associated with telerehabilitation programs. Risks included the security of patient data, patient privacy, or consultation from an unauthorized person. Additionally, 21% indicated that a patient may feel it is difficult to explain their condition to their doctor or therapist via telerehabilitation.

## DISCUSSION

Results of this study suggest that rehabilitation professionals believe that telerehabilitation can improve access to healthcare in Pakistan. While solutions to the shortage of rehabilitation centers and rehabilitation professionals in Pakistan require further study (Rathore et al., 2011), it is clear that a variety of services (i.e., ranging from consultation to therapy), can be offered through telerehabilitation (Marzano & Lubkina, 2017). Telerehabilitation services may be the best choice for in-home rehabilitation services, due to efficient delivery and cost effectiveness (Otsen & Agyei-Baffour, 2017).

The use of telerehabilitation could advance the Pakistani government’s achievement of the WHO Disability Objectives of 2021 by improving rural health care and the rehabilitation service delivery system nationwide. Telerehabilitation programs could incorporate primary healthcare strategies, such as promoting preventive measures. This could help the government increase the geographic coverage area and ease disease-related and economic burdens (Khan et al., 2017).

COMSATS is an ongoing telehealth program in Pakistan; however, it provides only consultation services in three practice areas: general medicine, dermatology and gynecology (Hussain, 2007). This program could be expanded and others initiated to offer a variety of services ranging from consultation to provision of different treatments and therapies (Gregory et al., 2011).

Almost all service delivery programs or treatment modalities carry some associated risks. While technological advancements are vital to the future of healthcare, new technologies can cause unintended, negative consequences to the lives and welfare of users. (Mishra, 2013). Previous researchers described potential risks associated with telerehabilitation; these are chiefly related to infringements on patient privacy and security (Watzlaf, Moeini, & Firouzan, 2010). The current study highlighted similar risks, including consultation from non-professionals. In addition to technology based challenges, low literacy and language related issues may pose vulnerabilities (Mishra, 2013).

## CONCLUSIONS

Our findings suggest that community based rehabilitation programs can be strengthened by using telerehabilitation, and that telerehabilitation can address the unserved needs of persons with disabilities located in remote areas.

It is encouraging that rehabilitation professionals in Pakistan self-assess as possessing sufficient knowledge about the ICT’s and telerehabilitation to employ their use. The data present positive indicators that professionals in Pakistan believe that they possess the technical and attitudinal readiness to use ICT and telerehabilitation to address nationwide shortages of rehabilitation facilities and personnel. It is important to recognize that professional training programs are often needed to upgrade the knowledge and experience level of professionals to meet any new program objectives (Movahedazarhouligh, Vameghi, Hatamizadeh, Bakhshi, & Mousavi Khatat, 2015).

We can therefore infer that the future of telerehabilitation in Pakistan is bright, but requires the attention of policy makers and non-government organizations to launch appropriate nationwide initiatives. The results of this study may be useful to policy makers (e.g., WHO, NGOs working in the rehabilitation sector, Health Ministry, and Ministry of Special Persons) as they assess the readiness of rehabilitation professionals to use telerehabilitation.

In conclusion, telerehabilitation has the potential to be cost effective and to be made readily available across Pakistan. The use of telehealth could empower the government to deliver enhanced primary health care and rehabilitation for a wide variety of services. It could also stimulate the acquisition of more specific and comprehensive data related to disabilities (and diseases) for policy making and budget allocation.

## Figures and Tables

**Table 1:** Characteristics of Participants

Gender Distribution	Frequency	Percentage
Female	152	52.9%
Male	135	47.1%
**Type of Phone Users**		
Smart Phone	278	97%
Simple Phone	9	3%
**Type of Professionals**		
Physiatrist	33	11.5%
Orthotist/Prosthetist	54	18.9%
Physical Therapist	79	27.6%
Psychologist	47	16.3%
Occupational Therapist	29	10.1%
Speech-Language Pathologist	38	13.2%
Rehabilitation Nurses	7	2.4%

**Table 2:** Survey Results

Knowledge about telemedicine	
Aware of technology but haven’t used it	41%
Aware of technology and use it on an intermittent basis	31%
Good knowledge of technology and use it on regular basis	14%
Don’t know about the technology	24%
**Knowledge about telerehabilitation**	
Aware of technology but haven’t used it	35%
Aware of technology and use it on an intermittent basis	34%
Good knowledge and use it on regular basis	9%
Don’t know about the technology	22%
**Methods to improve the rehabilitation services delivery system**	
Telerehabilitation	23%
Community Based Rehabilitation	38%
Both Telerehabilitation and Community Based Rehabilitation	39%
**Services that can be offered using telerehabilitation**	
Consultation, prescribing, delivery of required therapy, or monitoring only	23%
Multiple services including consultation, prescription, delivery of complex therapies, monitoring, evaluation, follow-ups and nursing care	68%
**Social acceptance of telerehabilitation in Pakistan**	
Extremely acceptable	21%
Very acceptable	35%
Somewhat acceptable	29%
Not acceptable	15%
**Expected limiting factors**	
Lack of ICT knowledge, high cost, rapidly changing ICT technology, attitude of policy makers, lack of skilled personnel and patient compliance	61%
Lack of ICT knowledge, high cost and rapidly changing ICT technology and patient compliance	27%
Lack of skilled personnel, attitude of policy maker and rapidly changing technology	22%
**Associated Risks**	
Patient data security, patient privacy, or consultation from unauthorized person	49%
Patient difficulty in explaining their conditions to the doctor or therapist	21%
Patient privacy and difficulty in developing relationship with doctor/therapist	13%
All of the above	17%
